# Association of epigenetic age acceleration with MRI biomarkers of aging and Alzheimer’s disease neurodegeneration

**DOI:** 10.18632/aging.206369

**Published:** 2026-04-07

**Authors:** Linda K. McEvoy, Bowei Zhang, Steve Nguyen, Adam X. Maihofer, Caroline M. Nievergelt, Ramon Casanova, Steve Horvath, Ake T. Lu, Christos Davatzikos, Guray Erus, Susan M. Resnick, Mark A. Espeland, Steve Rapp, Kenneth Beckman, Luigi Ferrucci, Andrea Z. LaCroix, Aladdin H. Shadyab

**Affiliations:** 1 Kaiser Permanente Washington Health Research Institute, Seattle, WA 98101, USA; 2 Herbert Wertheim School of Public Health and Human Longevity Science, University of California San Diego, La Jolla, CA 92037, USA; 3 Department of Psychiatry, School of Medicine, University of California San Diego, La Jolla, CA 92093, USA; 4 Veterans Affairs San Diego Healthcare System, Research Service, San Diego, CA 92161, USA; 5 Department of Biostatistics and Data Science, Wake Forest University School of Medicine, Winston-Salem, NC 27101, USA; 6 Altos Labs Cambridge Institute of Science, Cambridge, UK; 7 Altos Labs, San Diego, CA 92121, USA; 8 Department of Radiology, University of Pennsylvania School of Medicine, Philadelphia, PA 19104, USA; 9 Laboratory of Behavioral Neuroscience, National Institute on Aging, National Institutes of Health, Baltimore, MD 21224, USA; 10 Division of Gerontology and Geriatric Medicine, School of Medicine, Wake Forest University School of Medicine, Winston-Salem, NC 27101, USA; 11 Department of Psychiatry and Behavioral Medicine, Wake Forest University School of Medicine, Winston-Salem, NC 27103, USA; 12 Genomics Center, University of Minnesota, Minneapolis, MN 55455, USA; 13 Longitudinal Studies Section, Translational Gerontology Branch, National Institute on Aging, Baltimore, MD 21225, USA; 14 Division of Geriatrics, Gerontology, and Palliative Care, Department of Medicine, University of California San Diego, La Jolla, CA 92093, USA

**Keywords:** epigenetic clocks, brain age, biological aging, smoking, frontal lobe

## Abstract

Epigenetic clocks of biological aging have been associated with cognitive impairment and dementia. Less is known about whether they are associated with an older-appearing brain or with an atrophy pattern associated with dementia. We examined associations of five epigenetic clocks measured at baseline with the Spatial Pattern of Atrophy for Recognition of Brain Aging (SPARE-BA) and the Alzheimer’s Disease Pattern Similarity Score (AD-PS) derived from structural MRIs obtained an average of 8 years later among 1,196 older women. Using linear regression models adjusting for relevant covariates, we observed no associations between any epigenetic clock and accelerated brain aging based on SPARE-BA. We observed a significant association between AgeAccelGrim2 and AD-PS (β = 0.015; 95% CI 0.004 to 0.027; p = 0.01). This association appeared to be primarily driven by the association of a DNA methylation marker of smoking pack years with frontal and temporal lobe volumes. AgeAccelGrim2 was not associated with volumes in regions implicated in early AD (hippocampus and entorhinal cortex). Taken together with prior findings, these results suggest that measures of epigenetic and brain age acceleration capture different aspects of biological aging, and that AgeAccelGrim2 is predictive of neurodegenerative changes associated with smoking that increase risk of dementia.

## INTRODUCTION

Advancing age is associated with cognitive decline and increased risk for Alzheimer’s disease and related dementias (ADRD). Biological aging is heterogeneous, differing between people of the same chronological age and among tissue types within the same individual [1]. Epigenetic clocks have been evaluated as measures of biological aging. First generation epigenetic clocks were developed by assessing DNA methylation (DNAm) patterns predictive of chronological age [2–4]. Second generation clocks assessed DNAm patterns predictive of aging-related health outcomes or mortality [5–7]. A third-generation clock assessed DNAm patterns predictive of 20-year change in multiple organ systems to generate a DNAm biomarker of the pace of aging [8]. Faster epigenetic aging relative to chronological age, considered a marker of accelerated biological aging, has been referred to as epigenetic age acceleration [4].

Epigenetic age acceleration has been associated with poorer cognitive performance, steeper cognitive decline with age, and increased risk of dementia across several epigenetic clocks, even though these clocks were not trained on cognitive outcomes [9–15]. Less is known about whether epigenetic clocks are associated with structural magnetic resonance imaging (MRI) evidence of advanced brain aging or of age-related neurodegenerative disorders such as Alzheimer’s disease (AD).

Aging is accompanied by characteristic changes in brain structure that overlap with, but are distinct from, changes that precede AD dementia [16, 17]. Composite MRI indices have been created to reflect brain signatures of aging and of AD. The Spatial Pattern of Atrophy for Recognition of Brain Aging (SPARE-BA) is an index of brain aging that was derived through application of a high-dimensional pattern classification algorithm to differentiate structural MRI data of younger adults from that of older adults, yielding a predicted brain age score [18, 19]. Older brain-predicted age than chronological age is indicative of accelerated brain aging and is referred to here as SPARE-BA acceleration, or SPARE-BAA.

The AD Pattern Similarity Score (AD-PS), a composite index of the degree to which an individual’s structural MRI reflects that of individuals diagnosed with dementia, was derived by applying high dimensional machine learning to structural MRI measures of gray matter to discriminate scans of healthy older adults from scans of those with AD dementia using data from the Alzheimer’s Disease Neuroimaging Initiative [20]. Higher scores reflect greater similarity with the spatial volumetric MRI pattern observed in participants with AD. The AD-PS strongly predicted future dementia in independent cohorts, including in the Women’s Health Initiative Memory Study (WHIMS) [21, 22]. In WHIMS, the AD-PS showed an area under the curve of 0.89 for discriminating those who developed dementia from cognitively stable women [21, 22].

Greater knowledge of whether epigenetic measures of accelerated aging are associated with structural MRI brain signatures of aging or AD may inform on underlying biological mechanisms and provide more accessible biomarkers for brain changes in aging and AD. To this end, we examined whether epigenetic aging is associated with older brain-predicted age than chronological age or with an atrophy pattern indicative of increased AD risk an average of 8 years later, in a large cohort of community dwelling older women from the WHIMS. We examined five commonly used epigenetic clocks that have been previously examined in WHIMS in relation to cognitive outcomes [13, 15, 23], including two first-generation clocks, Horvath’s intrinsic epigenetic age acceleration (IEAA) and Hannum’s extrinsic epigenetic age acceleration (EEAA), two second-generation clocks, DNAm PhenoAge (AgeAccelPheno) [5] and GrimAge2 (AgeAccelGrim2) [6, 7], and a third-generation clock, Dunedin (P)ace of (A)ging (C)alculated from the (E)pigenome (DunedinPACE) [8].

## RESULTS

Several health-related characteristics differed by AgeAccelGrim2 quartiles, as shown in Supplementary Table 1. Women who reported never smoking were most likely to be in the lowest AgeAccelGrim2 quartile whereas current smokers were most likely to be in the highest quartile. Women in the lowest quartile of AgeAccelGrim2 were more likely to have higher levels of physical activity and lower body mass index (BMI), whereas those in the highest quartile were more likely to have prevalent diabetes. There were no differences across AgeAccelGrim2 quartiles by hormone treatment (HT) arm, race, ethnicity, education, APOE ε4 carrier status, cardiovascular disease (CVD), or non-melanoma cancer.

**Figure 1:**
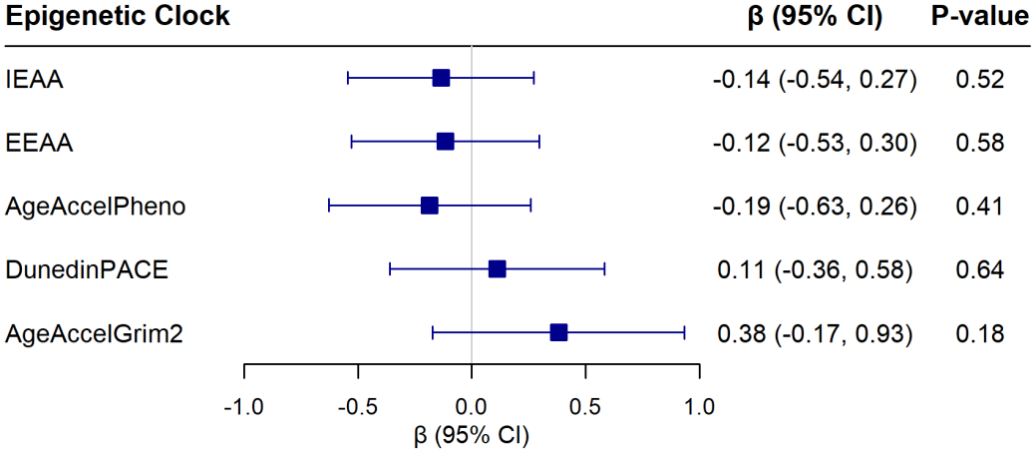
Associations of five epigenetic aging clocks with SPARE-BAA from linear regression models adjusting for chronological age, hormone therapy trial arm, education, smoking status, race, ethnicity, physical activity, BMI, diabetes, cardiovascular disease, cancer, and blood cell composition (models for IEAA and EEAA did not include blood cell composition). Beta coefficients are reported for each 1 standard deviation increase in the epigenetic measure.

None of the epigenetic clocks were significantly associated with SPARE-BAA, as indicated in Figure 1, which shows associations of epigenetic clocks with SPARE-BAA from fully adjusted models (see Supplementary Figure 1 for scatter plots). Minimally-adjusted models also showed non-significant associations (Supplementary Table 2).

**Figure 2:**
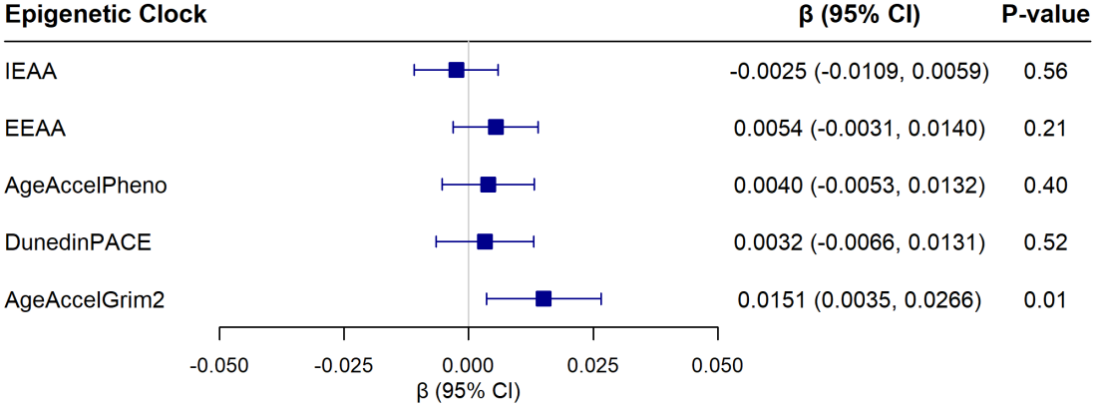
Associations of five epigenetic aging clocks with the Alzheimer’s Disease Pattern Similarity Score [ln (AD-PS + 1)], from linear regression models adjusting for chronological age, hormone therapy trial arm, education, smoking status, race, ethnicity, physical activity, BMI, diabetes, cardiovascular disease, cancer, and blood cell composition (models for IEAA and EEAA did not include blood cell composition). Beta coefficients are reported for each 1 standard deviation increase in the epigenetic measure.

**Figure 3:**
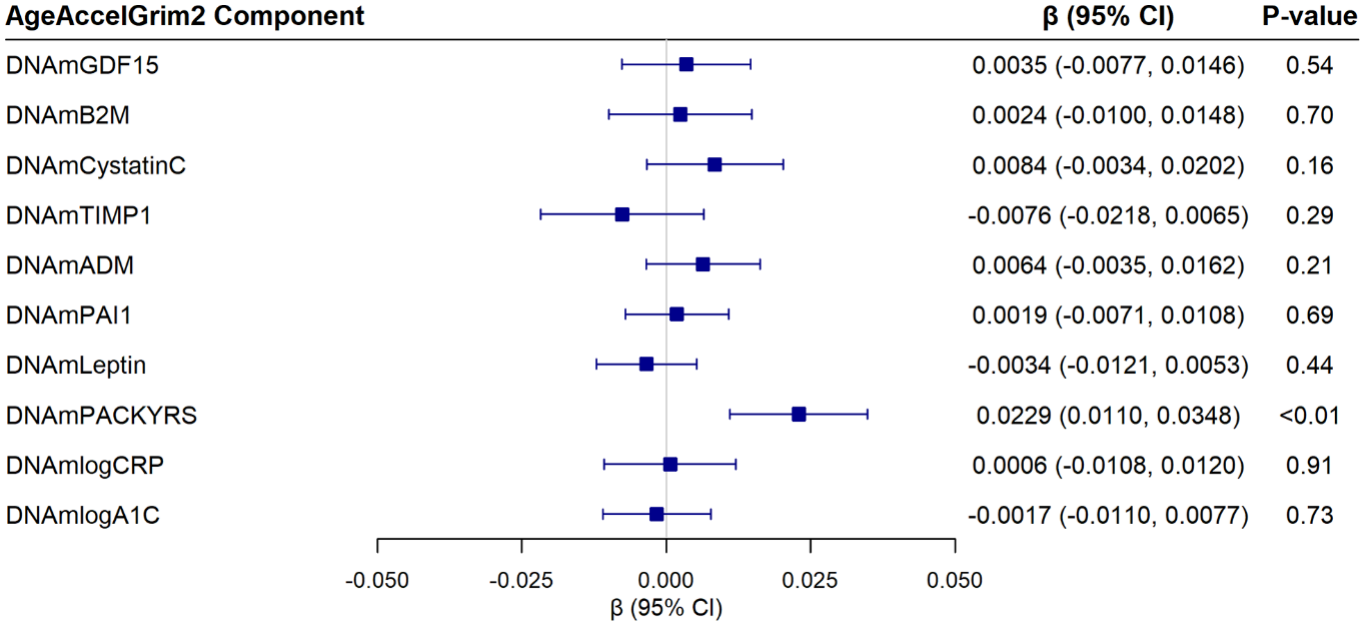
Associations of the ten epigenetic components of the AgeAccelGrim2 clock with the AD-PS score from linear regression models adjusting for chronological age, hormone therapy trial arm, education, smoking status, race, ethnicity, physical activity, BMI, diabetes, cardiovascular disease, cancer, and blood cell composition. Beta coefficients are reported for each 1 standard deviation increase in the epigenetic measure. Abbreviations: DNAm GDF-15: epigenetic marker of growth differentiation factor 15; DNAm B2M: epigenetic marker of beta-2 microglobulin; DNAm Cystatin C: epigenetic marker of cystatin C; DNAm TIMP-1: epigenetic marker of tissue inhibitor metalloproteinase 1; DNAm ADM: epigenetic marker of adrenomedullin; DNAm PAI-1: epigenetic marker of plasminogen activation inhibitor 1; DNAm Leptin: epigenetic marker of leptin; DNAm Packyrs: epigenetic marker of smoking pack-years; DNAm logCRP: epigenetic marker of log-scale high sensitivity C-reactive protein; DNAm logA1C: epigenetic marker of log hemoglobin A1C.

Of the five epigenetic clocks examined, only AgeAccelGrim2 was significantly associated with the AD-PS score (β = 0.015; 95% CI 0.004 to 0.027; p = 0.01), an association that survived correction for multiple comparisons (see Figure 2 for results from fully adjusted models for each of the clocks, Supplementary Table 3 for results of progressively adjusted models, and Supplementary Figure 2 for scatter plots). Each one standard deviation increase in AgeAccelGrim2 (4.15) was associated with a 1.5% higher ln(AD-PS+1) score. Of the 10 components of AgeAccelGrim2, only the DNAm marker of smoking pack years was associated with the ln(AD-PS+1) score (β = 0.023; 95% CI 0.011 to 0.035; p < 0.01) (Figure 3). These associations remained significant after further adjustment for smoking pack years based on self-report (AgeAccelGrim2: β = 0.013; 95% CI 0.001 to 0.025; p = 0.04; DNAm smoking pack years: 0.022; 95% CI 0.009 to 0.035; p < 0.01) and after adjustment for alcohol use (AgeAccelGrim2: β = 0.014; 95% CI 0.003 to 0.026; p = 0.01; DNAm smoking pack years: 0.023; 95% CI 0.011 to 0.035; p < 0.01).

**Figure 4:**
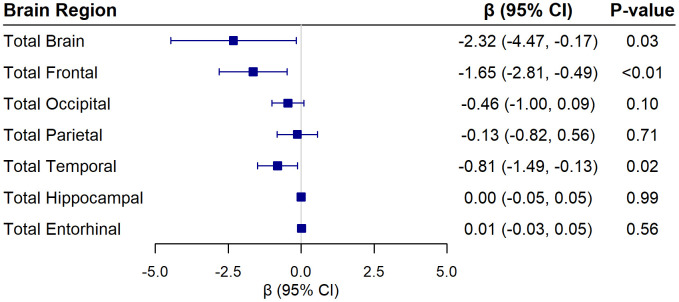
Associations of AgeAccelGrim2 with global and regional brain volumes (in cm^3^) from linear regression models adjusting for chronological age, hormone therapy trial arm, education, smoking status, race, ethnicity, physical activity, BMI, diabetes, cardiovascular disease, cancer, blood cell composition, and intracranial volume. Beta coefficients are reported for each 1 standard deviation increase in the epigenetic measure.

**Figure 5:**
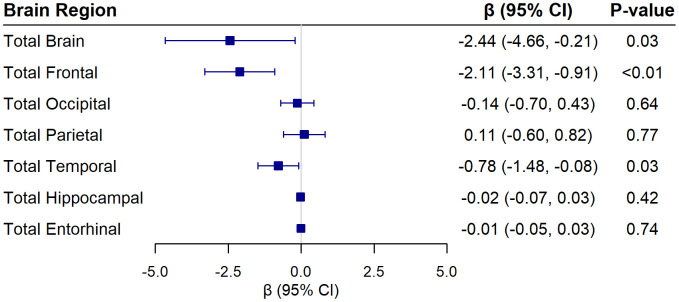
Associations of DNAm SmokingPackYears from AgeAccelGrim2 with global and regional brain volumes (in cm^3^) from linear regression models adjusting for chronological age, hormone therapy trial arm, education, smoking status, race, ethnicity, physical activity, BMI, diabetes, cardiovascular disease, cancer, blood cell composition, and intracranial volume. Beta coefficients are reported for each 1 standard deviation increase in the epigenetic measure.

In secondary analyses examining associations of AgeAccelGrim2 with regional brain volumes, AgeAccelGrim2 was inversely associated with total brain volume (β = -2.32; 95% CI -4.47 to -0.17; p = 0.03), frontal lobe volume (β = -1.65; 95% CI -2.81 to -0.49; p<0.01) and temporal lobe volume (β = -0.81; 95% CI -1.49 to -0.13; p = 0.02). There were no associations with other brain regions, including hippocampus and entorhinal cortex (Figure 4). The same pattern of results was observed with DNAm smoking pack-years as the exposure (Figure 5).

The exclusion of 46 women with MCI or dementia at time of MRI visit did not change the results (see Supplementary Table 4 and Supplementary Figure 3). AgeAccelGrim2 continued to show a significant association with the ln(AD-PS+1) score (β = 0.016; 95% CI 0.005 to 0.028; p < 0.01) in these analyses. Associations of AgeAccelGrim2 with the ln(AD-PS+1) and SPARE-BAA did not differ by APOE ε4 status (all *P*-values for interaction > 0.10 in the fully adjusted models; see Supplementary Tables 5, 6). Adjustment for time between blood draw and MRI scan did not affect the results (Supplementary Tables 7, 8).

## DISCUSSION

Leveraging epigenetic and neuroimaging data from a large, diverse cohort of older women from WHIMS, we examined whether accelerated biological aging as indicated by five well-studied epigenetic clocks was associated with accelerated brain aging or with an AD-pattern of brain atrophy from MRIs obtained 8 years later. We found that none of the epigenetic clocks were associated with accelerated brain aging as measured by the SPARE-BAA. AgeAccelGrim2 was associated with higher AD-PS score, a structural MRI index predictive of dementia. Exploratory analyses of associations of 10 individual components of this clock with the AD-PS score, and with individual brain regions, suggested that the association of AgeAccelGrim2 with the AD-PS score was driven by the association of one component, the epigenetic biomarker of smoking pack years, with reduced frontal and temporal lobe volumes.

Relatively few studies have compared epigenetic clocks with measures of brain age acceleration from structural MRI, and those that have, have generally reported no or weak associations [24]. Across studies, AgeAccelGrim was most likely to correlate with accelerated brain aging [24]. For example, there were no significant associations of IEAA, EEAA, or AgeAccelPheno with SPARE-BAA among 326 middle-aged adults (mean age 50 and 55 years at time of MRI) in the CARDIA study, but AgeAccelGrim was weakly and positively associated with SPARE-BAA measured 10 (r=.16) and 15 (r=.18) years later [25]. Using Cole’s brainAgeR method to predict age from structural MRI data, AgeAccelGrim but not AgeAccelHorvath was positively associated with the brain-predicted age difference (brain-PAD; calculated by subtracting chronological age from predicted brain age) [26, 27]. In contrast, accelerated biological aging was not associated with brain-PAD among 560 men and women aged ≥ 70 years in the ASPREE study for any of the epigenetic clocks examined (Horvath, Hannum, PhenoAge, DunedinPACE, GrimAge, or GrimAge2) [28]. Ferreira et al. reported significant associations of AgeAccelHannum and AgeAccelGrim with brain-PAD among 254 adults with a wide age range (20 – 84 years); AgeAccelHorvath, AgeAccelPheno, or DunedinPACE were not associated with brain-PAD [24]. SPARE-BAA and brain-PAD have been associated with many of the same aging-related health outcomes and mortality as epigenetic clocks [18, 27]. The general lack of correlation of epigenetic and brain age acceleration measures suggests that these metrics capture different aspects of biological aging.

In contrast to the lack of association of any of the epigenetic clocks with SPARE-BAA, we observed a significant association of AgeAccelGrim2 with the AD-PS score: increasing AgeAccelGrim2 was associated with greater similarity to a volumetric pattern predictive of dementia. This is consistent with the results of our prior study among WHIMS women, which showed that, of five epigenetic clocks examined, only AgeAccelGrim2 was significantly associated with incident MCI/dementia after multiple comparison correction [23].

Secondary analyses examining associations of AgeAccelGrim2 with total and regional structural brain measures indicated that AgeAccelGrim2 was associated with lower total brain volume, lower frontal lobe volume, and to a lesser extent, lower temporal lobe volume. AgeAccelGrim2 was not associated with hippocampal or entorhinal volume, brain regions that are strongly implicated in early AD. This may suggest that the association of AgeAccelGrim2 with the AD-PS score may not reflect primarily AD-related pathology.

The GrimAge2 clock predicts biological age based on chronological age, sex, and 10 DNAm-based surrogates of plasma proteins, including an epigenetic signature of smoking pack years. Of these, the DNAm surrogate of smoking pack years showed similar associations as AgeAccelGrim2 with AD-PS and with total brain, frontal and temporal volumes, and similar lack of association with hippocampal and entorhinal cortex volumes.

In the Lothian Birth Cohort, a sample that includes both men and women, AgeAccelGrim2 and DNAm smoking pack years were also associated with total brain volume, as well as frontal and temporal lobe volumes [29]. In that study, effect sizes for the cross-sectional association of AgeAccelGrim2 with brain outcomes were larger than for DNAm smoking packyears, suggesting that other plasma proteins contributed to AgeAccelGrim2 associations with brain outcomes. In our study, associations of DNAm smoking packyears with brain outcomes 8 years later were nominally larger than for AgeAccelGrim2, suggesting that the epigenetic marker of smoking underlies much of the association of AgeAccelGrim2 with AD-PS in older women.

While the number of current smokers in our sample at baseline was very small (less than 5%), there were many former smokers (39%). Epigenetic changes related to smoking have been shown to persist for 30 years after smoking cessation [30]. Furthermore, the epigenetic marker of smoking pack years has been shown to be more strongly predictive of mortality than smoking pack years based on self-report and has been shown to be associated with mortality even among non-smokers [6]. This marker may thus reflect epigenetic changes related to exposure to second-hand smoke or to other environmental toxins. Consistent with this, we found that associations remained significant even after adjustment for smoking pack years.

Smoking is a strong cardiovascular risk factor and is associated with increased risk of dementia [31]. Smoking is known to be associated with accelerated brain aging and with reduced brain volume, particularly in the frontal cortex [32–37]. Taken together, the lack of association of AgeAccelGrim2 with hippocampus and entorhinal cortex volumes, and the strong association with frontal cortex volume that appears to be driven by smoking-related epigenetic changes, suggests that AgeAccelGrim2 may be more predictive of vascular contributions to dementia and to AD pathology. In support of this, we recently observed that AgeAccelGrim2 was associated with a non-specific plasma biomarker of neural injury, neurofilament light chain protein (NfL) at baseline, but not with biomarkers of amyloid pathology, phosphorylated tau at threonine-181 (p-tau181) or phosphorylated tau at threonine-217 (ptau 217) at baseline [38].

Our study has several strengths, including a large sample of older women, the prospective design examining baseline epigenetic clocks in relation to brain outcomes ~8 years later, and the ability to examine five epigenetic clocks and two validated composite structural neuroimaging measures created to predict brain aging and Alzheimer’s dementia risk. We were able to adjust for many potentially confounding sociodemographic, behavioral, and health variables, and were able to explore associations with regional brain measures in addition to the composite scores.

Our study has several limitations. The sample was restricted to women aged 65 years and older and had limited racial and ethnic diversity. Therefore, results may not generalize to men, younger women, or to samples with greater diversity. Associations of epigenetic clocks with predicted brain age may differ in samples that include a larger age range. We also examined only a single MRI signature of brain aging, SPARE-BAA; results may differ for other structural brain age measures. Similarly, results may differ for other neuroimaging modalities of predicted brain age and AD neuroimaging signatures. Our analyses were limited to five commonly examined epigenetic clocks that have been previously examined in WHIMS in relation to cognitive outcomes [13, 15, 23]. Future research using newer epigenetic clocks, and those developed to predict brain or cognitive outcomes, is warranted.

In summary, our study suggests that none of the five most commonly assessed epigenetic clocks are significantly associated with a measure of accelerated brain aging derived from structural MRIs. We found that AgeAccelGrim2 was associated with the AD-PS score, a volumetric pattern reflective of increased risk of dementia. This association appeared to be driven primarily by smoking-related differences in the frontal and temporal lobes and likely does not reflect processes specific to AD. Together with our prior findings of the association of AgeAccelGrim2 with incident MCI/dementia [23], and with a plasma biomarker of neural injury [38], these findings underscore the robustness of AgeAccelGrim2 as a predictor of cognitive impairment and neurodegeneration.

## MATERIALS AND METHODS

### Study population

WHIMS is an ancillary study of the Women’s Health Initiative (WHI) hormone therapy trials. WHIMS was designed to investigate the effects of hormone therapy on cognitive outcomes among 7,479 postmenopausal women ages 65-80 years who were cognitively unimpaired at randomization in 1995-1998. Details on the WHIMS design and protocols have been published [39]. Women were recruited across 39 U.S. clinical centers and randomized to either conjugated equine estrogen (CEE) vs. matching placebo or CEE with medroxyprogesterone acetate (CEE+MPA) vs. matching placebo. Annual in person follow-up for cognitive outcomes continued through 2007. In 2008, WHIMS transitioned to annual telephone-administered cognitive assessments in the WHIMS Epidemiology of Cognitive Health Outcomes (WHIMS-ECHO) study, which followed participants for cognitive outcomes through 2021 [40]. Among 7,479 WHIMS participants, we excluded 240 with only one WHIMS cognitive assessment, 519 who did not consent to data sharing of their genetic data through dbGaP, and 304 with no baseline DNA or buffy coat available, leaving 6,416 participants whose baseline biospecimens were used for epigenetics measurement. After quality control (see Supplementary Methods), epigenetic data were available for 6,069 participants. Of these, 1,196 women had available brain MRI data.

### Epigenetic clocks

DNA methylation was measured in whole blood, collected at WHI baseline, at the University of Minnesota Genomics Center using the Illumina EPIC v2 BeadChip (Illumina, Inc., San Diego, CA, USA), which measures DNA methylation at ~930,000 cytosine-guanine dinucleotide (CpG) sites. Details of the preprocessing and quality control steps for derivation of epigenetic data are reported in the Supplementary Methods.

We examined five commonly used epigenetic clocks, including intrinsic epigenetic age acceleration (IEAA), a version of the Horvath clock that measures cell-intrinsic methylation changes controlling for age-related differences in blood cell composition, and extrinsic epigenetic age acceleration (EEAA) based on the Hannum clock that incorporates age-related differences in white blood cell-type composition. [41] The Horvath and Hannum clocks are first-generation epigenetic clocks that were trained to predict chronological age. We also examined two second-generation clocks that were trained to predict an aging-related clinical phenotype or mortality. These included DNAm PhenoAge (AgeAccelPheno), which was created by regressing DNAm patterns on a composite clinical phenotype that included biomarkers of kidney, liver, metabolic, and immune function, as well as chronological age [5]; and GrimAge, which was created by regressing a composite biomarker of nine DNAm surrogates of health-related plasma proteins, a DNAm-based estimator of smoking pack-years, age, and sex on mortality risk [6, 7]. A second version of this clock, GrimAge2, additionally incorporated DNAm measures of log-scale C-reactive protein for inflammation and log-scale hemoglobin A1C for glucose metabolism [7]. We used GrimAge2 in our analyses. We also included a third-generation clock, DunedinPACE, which was developed to predict longitudinal change in biomarkers of cardiovascular, metabolic, renal, hepatic, immune, dental, and pulmonary systems among middle-aged adults followed for 20 years [8]. Age acceleration from first- and second-generation clocks was measured as the residual from regressing epigenetic age on chronological age. The online Horvath and Clock Foundation DNAm Age Calculator (https://dnamage.clockfoundation.org/) was used to calculate IEAA, EEAA, AgeAccelPheno, and AgeAccelGrim2. DunedinPACE was calculated using R code available at https://github.com/danbelsky/DunedinPACE.

In secondary analyses, we examined the individual DNAm-based components of AgeAccelGrim2: smoking pack-years (DNAm Packyrs), adrenomedullin (DNAm ADM), beta-2 microglobulin (DNAm B2M), cystatin C (DNAm Cystatin C), growth differentiation factor 15 (DNAm GDF-15), leptin (DNAm Leptin), log-scale high sensitivity C-reactive protein (DNAm logCRP), log-scale hemoglobin A1C (DNAm logA1C), plasminogen activation inhibitor 1 (DNAm PAI-1), and tissue inhibitor metalloproteinase 1 (DNAm TIMP-1) [7].

For interpretation purposes, all epigenetic clock variables were standardized with a mean of 0 and SD of 1. Higher values of first- and second-generation epigenetic clocks indicate accelerated biological aging relative to chronological age, whereas lower values indicate slower biological aging. DunedinPACE measures the pace of biological aging. DunedinPACE values >1 indicate faster pace of aging (e.g., a value of 1.10 indicates a pace of aging 10% faster than the norm for adults in midlife), while values <1 indicate slower pace of aging (e.g., a value of 0.90 indicates a pace of aging 10% slower than the norm for adults in midlife).

### Brain MRI outcomes

The WHIMS-MRI study was designed to compare MRI findings among women assigned to the intervention vs. placebo arms in the WHIMS trial [42, 43]. WHIMS-MRI was conducted in 14 of the 39 clinical centers that participated in WHIMS during 2005-2006. Details of the MRI protocol, and differences between MRI participants and non-participants have been described [42, 43]. The average time from WHIMS baseline (i.e., the time point of epigenetics measurement) to the MRI scan was 7.97 (0.59) years (range of 6.43 to 10.20 years).

MRI scans were acquired using a standardized scanning protocol developed by the MRI Quality Control Center in the Department of Radiology, University Pennsylvania, described in detail elsewhere [42, 43]. Briefly, scans were obtained with a 22 cm field of view and a matrix of 256 x 256 in 1.5T scanners and included oblique axial spin density/T2-weighted spin echo (TR:3200 ms, TE=30/120 ms, slice thickness= 3 mm), fluid-attenuated inversion recovery (FLAIR) T2-weighted spin echo (TR=8000 ms, TI=2000 ms, TE=100 ms, slice thickness=3 mm), and oblique axial three-dimensional T1-weighted gradient echo (flip angle=30 degrees, TR=21 ms, TE=8 ms, slice thickness=1.5 mm) images from the vertex to the skull base parallel to the anterior commissure-posterior commissure (AC-PC) plane [21].

A fully automated processing pipeline was applied to each participant’s T1-weighted scan, as previously described [19, 44, 45]. Preprocessing included correction of magnetic field intensity inhomogeneity and skull-stripping. Each T1 scan was segmented into pre-defined anatomical regions of interest (ROIs) using a multi-atlas, multi-warp label-fusion method, MUSE (MULti-atlas region Segmentation utilizing Ensembles). In the MUSE framework, multiple atlases with semi-automatically extracted ground-truth ROI labels are first warped individually to the target image using two different non-linear registration methods. A spatially adaptive weighted voting strategy is then applied to fuse the ensemble into a final segmentation. Each image was segmented into 145 ROIs spanning the whole brain. Using these data, a summary signature of brain aging, the SPARE-BA index, was derived through application of a high-dimensional pattern classification algorithm to differentiate structural MRI data of younger adults from that of older adults, as previously described [18, 19]. We regressed the SPARE-BA score on chronological age at time of the MRI scan to derive a measure of brain-predicted age difference, in which higher brain-predicted age than chronological age is indicative of accelerated brain aging, which has been referred to as SPARE-BA acceleration, or SPARE-BAA [25].

AD-PS scores were derived by applying high-dimensional machine learning to structural MRI measures of gray matter to discriminate AD scans from those of healthy controls, as previously described [20, 21]. Higher scores reflect greater similarity of an individual’s MRI volumetric pattern to that found in individuals with AD. Given its non-normal distribution, the AD-PS score was natural log-transformed prior to analysis, after adding 1 to avoid values of zero.

Our two primary outcomes were the SPARE-BAA and ln(AD-PS score +1). We examined total and regional brain volumes (cm^3^) including frontal, temporal, parietal, and occipital volumes, as well as hippocampus and entorhinal cortex, as secondary outcomes.

### Covariates

Baseline questionnaires assessed age, race (American Indian/Alaskan Native, Asian, Native Hawaiian or other Pacific Islander, Black, White, more than once race, or unknown/not reported), ethnicity (Hispanic/Latino, not Hispanic/Latino, or unknown/not reported), education (less than high school equivalent, high school diploma or GED, some college, college graduate), smoking status (never smoked, past smoker, current smoker), alcohol use (never consumed alcohol, past use, current light use with up to 7 drinks/week, and heavier use with more than 7 drinks per week); treated diabetes, cardiovascular disease, cancer, and total energy expenditure from recreational physical activity (in MET-hours/week). Height and weight were measured with a stadiometer and balance beam scale, respectively, to calculate BMI. *APOE* ε4 carrier status, defined as presence of at least 1 ε4 allele, was determined in women with available genome-wide genotyping data based on 2 single nucleotide polymorphisms (SNPs), rs429358 and rs7412. These data were available for White women only (n= 1049). Imputation was performed using the 1000 Genomics Project reference panel and the MaCH algorithm implemented in Minimac.[46] Both SNPs had high imputation quality (R^2^ >0.97 for rs429358 and R^2^>0.97 for rs7412) [15]. Hormone therapy treatment arm (estrogen alone, estrogen placebo, estrogen plus progestin, or estrogen plus progestin placebo) was also included as a covariate. White blood cell (WBC) counts were estimated for CD4+ T cells, CD8+ T cells, natural killer cells, B cells, monocytes, and neutrophils using IDOL [47]. For examination of secondary MRI outcomes of total and regional volumes, we additionally adjusted for estimated total intracranial volume.

### Statistical analysis

Because we previously found that AgeAccelGrim2 was more strongly associated with MCI/dementia in WHIMS than any of the other epigenetic clocks [23], we examined baseline characteristics by quartiles of AgeAccelGrim2. Differences among quartiles were tested using the Kruskal-Wallis rank sum test for continuous variables, Pearson’s chi-square test of independence for categorical variables, and Fisher’s exact test with simulated p-value (based on 2000 replicates) for categorical variables with low expected counts (e.g. race, ethnicity).

To examine the associations of epigenetic clocks with the primary outcomes, and with total or regional volumes in secondary analyses, we conducted multivariable linear regression models to generate beta estimates and their 95% CIs for 1 SD differences in the epigenetic clock measure. Separate models were fit for each epigenetic clock and each MRI outcome. We describe progressively adjusted models. The minimally adjusted models controlled for chronological age (model 1) as well as race (White, Black and other) and Hispanic ethnicity (yes/no) (model 2). The next model additionally adjusted for hormone therapy trial arm, education, and smoking status (model 3). The full model additionally adjusted for physical activity, BMI, diabetes, cardiovascular disease, cancer, and blood cell composition. Models for IEAA and EEAA did not control for blood cell composition because IEAA was developed to be independent of blood cell composition and EEAA tracks age-related changes in blood cell composition [41]. In examination of global and regional brain volumes in secondary analyses, intracranial volume was also included as a covariate. We also examined individual components of AgeAccelGrim2 in secondary analyses.

There were very little missing data for covariates used in the main analyses (<1%; see Supplementary Table 1). Missing covariate data were imputed using multivariate imputation by chained equations using the R *mice* package, specifying all study variables with 20 imputations and 20 iterations.

In sensitivity analyses, we further adjusted for smoking pack years and alcohol use. We also examined associations after excluding 46 women who were diagnosed with MCI or probable dementia prior to the MRI scan. We examined effect modification by *APOE* ε4 carrier status by examining interaction terms of epigenetic clocks and *APOE* ε4 in the models; the significance of the interactions was examined using likelihood ratio tests. Finally, we reran the models for the primary outcomes SPARE-BAA and AD-PS including time between blood draw and MRI scan as a covariate in all progressively adjusted models.

To account for multiple testing across five clocks, we applied a Bonferroni-corrected threshold of *P≤*0.01 (0.05/5) for statistical significance for each primary outcome (SPARE-BAA and ln(AD-PS+1)). We report uncorrected p values and interpret the findings for primary outcomes based on this Bonferroni-corrected threshold. We did not consider multiple correction for p-values in secondary analyses, as these analyses were considered exploratory. All analyses were conducted using R statistical software version 4.4.2.

## Supplementary Material

Supplementary Methods

Supplementary Figures

Supplementary Table 1

Supplementary Tables 2-8
