# Supplementary material for: Association of epigenetic age acceleration with MRI biomarkers of aging and Alzheimer’s disease neurodegeneration
Source: Aging (Albany NY). 2026 Apr 7;18(1):303–26. doi: 10.18632/aging.206369 (PMC13285947; doi:10.18632/aging.206369)
Supplement: Supplementary Figures [file aging-18-1-206369-s002.pdf]

SUPPLEMENTARY FIGURES

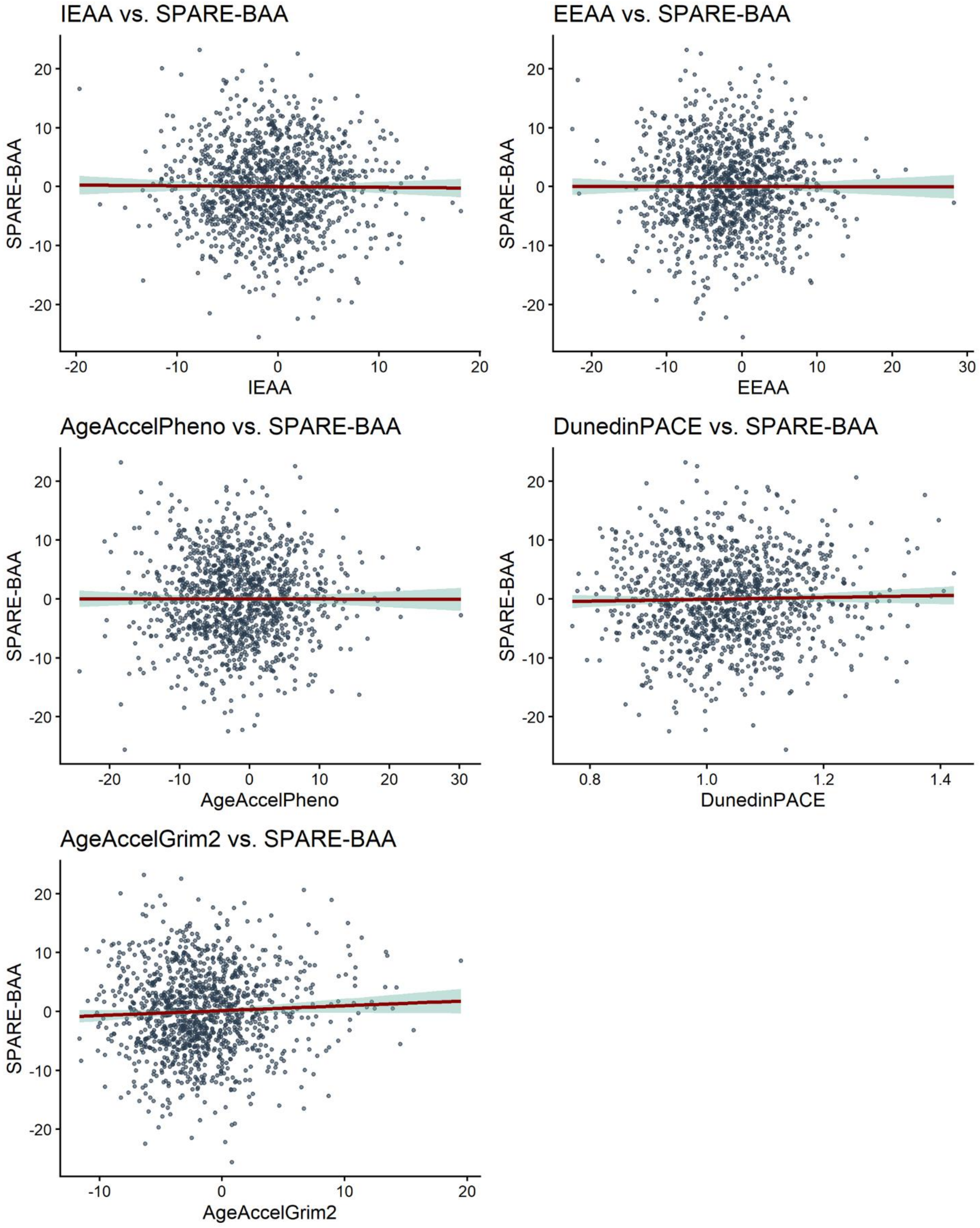

Supplementary Figure 1. Scatter plots of the association of SPARE- BAA with each of the five epigenetic clocks.

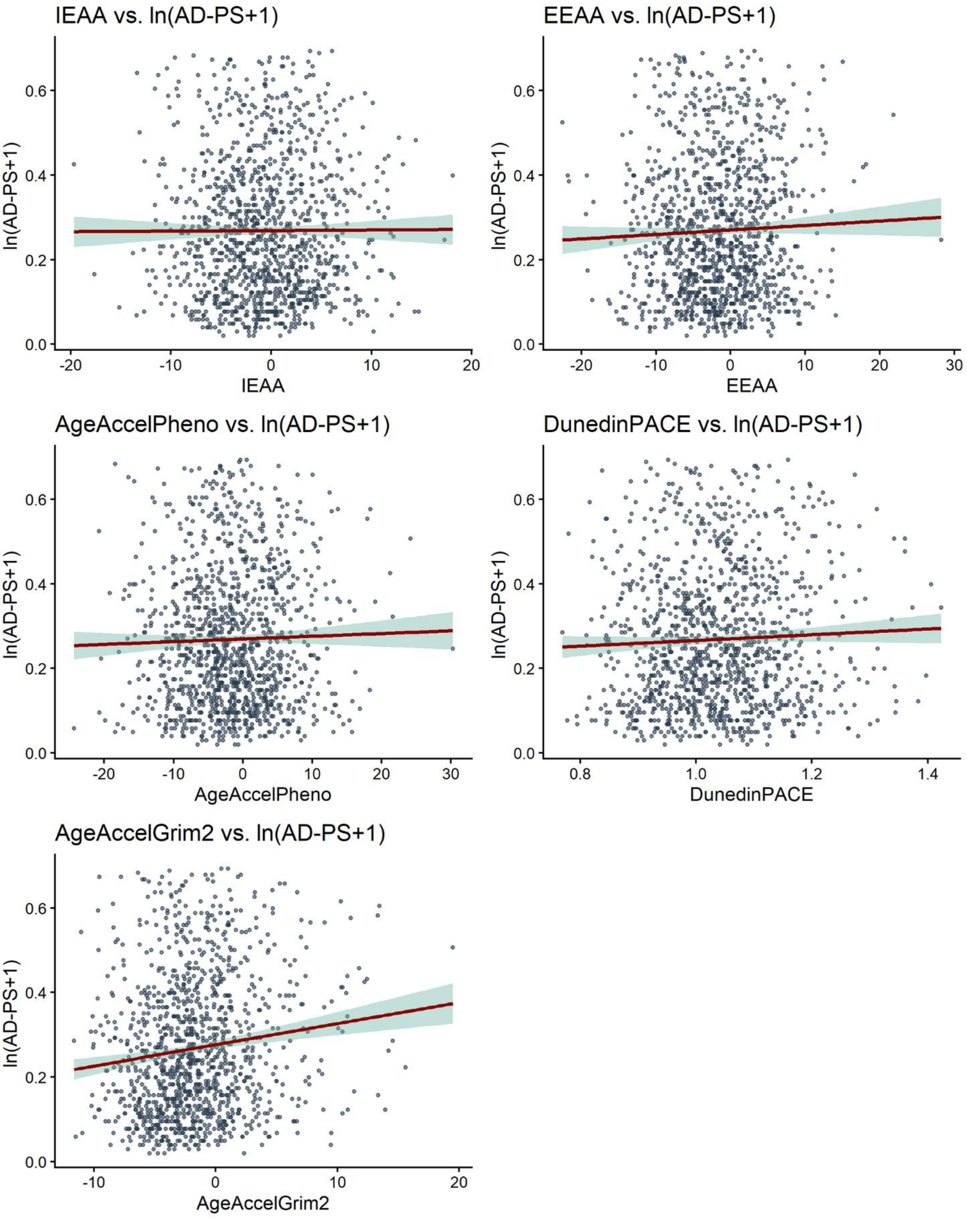

Supplementary Figure 2. Scatter plots of the association of ln(AD-PS + 1) with each of the five epigenetic clocks.

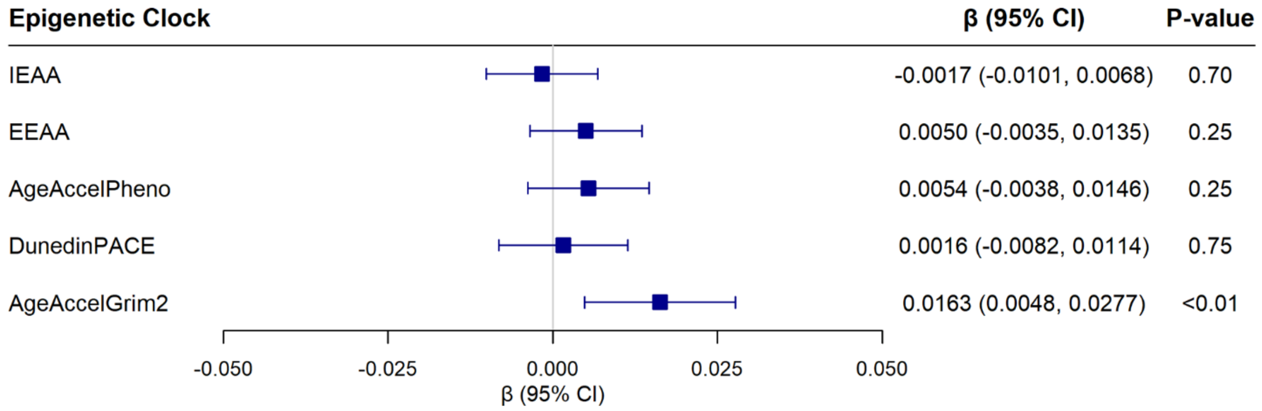

Supplementary Figure 3. Associations of the five epigenetic aging clocks with ln(AD-PS + 1), excluding 46 women who were diagnosed with MCI or AD prior to the MRI scan from linear regression models adjusting for chronological age, hormone therapy trial arm, education, smoking status, race, and ethnicity, physical activity, BMI, diabetes, cardiovascular disease, cancer, and blood cell composition (models for IEAA and EEAA did not include blood cell composition).
