# Supplementary material for: Association of epigenetic age acceleration with MRI biomarkers of aging and Alzheimer’s disease neurodegeneration
Source: Aging (Albany NY). 2026 Apr 7;18(1):303–26. doi: 10.18632/aging.206369 (PMC13285947; doi:10.18632/aging.206369)
Supplement: Supplementary Table 1 [file aging-18-1-206369-s003.docx]

**Supplementary Table 1. Demographic and clinical characteristics by quartile of AgeAccelGrim2.**

| **AgeAccelGrim2** | **Overall N = 1,196** | **Q1  [-11.64, -4.35] N = 297** | **Q2  [-4.35, -2.07] N = 301** | **Q3  [-2.07, 0.57] N = 298** | **Q4 [0.57, 19.49] N = 300** | **p-value** |
| --- | --- | --- | --- | --- | --- | --- |
| **Age, Mean (SD)** | 70 (4) | 69 (3) | 70 (4) | 69 (3) | 70 (4) | 0.003 |
| **Hormone Therapy arm, n (%)** |  |  |  |  |  | 0.2 |
| Estrogen-alone intervention | 228 (19%) | 48 (16%) | 56 (19%) | 71 (24%) | 53 (18%) |  |
| Estrogen-alone placebo | 213 (18%) | 48 (16%) | 53 (18%) | 45 (15%) | 67 (22%) |  |
| Estrogen+Progestin intervention | 365 (31%) | 98 (33%) | 92 (31%) | 87 (29%) | 88 (29%) |  |
| Estrogen+Progestin placebo | 390 (33%) | 103 (35%) | 100 (33%) | 95 (32%) | 92 (31%) |  |
| **Education, n (%)** |  |  |  |  |  | 0.14 |
| Less than high school equivalent | 53 (4.4%) | 11 (3.7%) | 20 (6.6%) | 11 (3.7%) | 11 (3.7%) |  |
| High school diploma or GED | 276 (23%) | 65 (22%) | 63 (21%) | 83 (28%) | 65 (22%) |  |
| Vocational, training school, Some college or associate degree | 469 (39%) | 116 (39%) | 131 (44%) | 106 (36%) | 116 (39%) |  |
| College graduate or higher | 395 (33%) | 104 (35%) | 87 (29%) | 97 (33%) | 107 (36%) |  |
| Missing | 3 | 1 | 0 | 1 | 1 |  |
| **Smoking, n (%)** |  |  |  |  |  | <0.001 |
| Never Smoked | 678 (57%) | 209 (71%) | 202 (67%) | 165 (56%) | 102 (34%) |  |
| Past Smoker | 457 (39%) | 85 (29%) | 96 (32%) | 125 (43%) | 151 (51%) |  |
| Current Smoker | 51 (4.3%) | 0 (0%) | 2 (0.7%) | 4 (1.4%) | 45 (15%) |  |
| Missing | 10 | 3 | 1 | 4 | 2 |  |
| **Smoking, pack years**, Mean (SD) | 8.7 (18.0) | 2.6 (7.5) | 4.9 (14.8) | 7.6 (15.5) | 19.3 (24.9) | <0.001 |
| Missing | 37 | 9 | 11 | 9 | 8 |  |
| **Alcohol consumption, n (%)** |  |  |  |  |  | 0.001 |
| Never | 146 (12.3%) | 38 (12.9%) | 40 (13.4%) | 43 (14.5%) | 25 (8.4%) |  |
| Past | 201 (16.9%) | 27 (9.2%) | 53 (17.7%) | 54 (18.2%) | 67 (22.5%) |  |
| Current light-moderate | 704 (59.3%) | 185 (62.9%) | 174 (58.2%) | 173 (58.2%) | 172 (57.7%) |  |
| Current heavy | 137 (11.5%) | 44 (15.0%) | 32 (10.7%) | 27 (9.1%) | 34 (11.4%) |  |
| Missing | 8 | 3 | 2 | 1 | 2 |  |
| **Race,** n (%) |  |  |  |  |  | 0.4 |
| American Indian/ Alaskan Native | 2 (0.2%) | 0 (0%) | 2 (0.7%) | 0 (0%) | 0 (0%) |  |
| Asian | 21 (1.8%) | 6 (2.0%) | 6 (2.0%) | 4 (1.4%) | 5 (1.7%) |  |
| Black | 55 (4.6%) | 9 (3.0%) | 11 (3.7%) | 14 (4.7%) | 21 (7.0%) |  |
| White | 1,104 (93%) | 279 (94%) | 278 (93%) | 277 (94%) | 270 (91%) |  |
| More than one race | 9 (0.8%) | 3 (1.0%) | 3 (1.0%) | 1 (0.3%) | 2 (0.7%) |  |
| Unknown or not reported | 5 | 0 | 1 | 2 | 2 |  |
| **Ethnicity,** n (%) |  |  |  |  |  | 0.3 |
| Not Hispanic or Latino | 1,171 (98%) | 293 (99%) | 296 (98%) | 291 (98%) | 291 (97%) |  |
| Hispanic or Latino | 24 (2.0%) | 3 (1.0%) | 5 (1.7%) | 7 (2.3%) | 9 (3.0%) |  |
| Unknown or not reported | 1 | 1 | 0 | 0 | 0 |  |
| **APOE e4 carrier status,** n (%) |  |  |  |  |  | 0.3 |
| No e4 alleles | 822 (78%) | 204 (74%) | 213 (80%) | 205 (78%) | 200 (80%) |  |
| At least one e4 allele | 230 (22%) | 70 (26%) | 52 (20%) | 57 (22%) | 51 (20%) |  |
| Missing | 144 | 23 | 36 | 36 | 49 |  |
| **Energy expenditure** (recreational physical activity, MET-hours/wk, Mean, SD) | 11 (13) | 13 (12) | 12 (14) | 10 (12) | 10 (13) | <0.001 |
| Missing | 2 | 0 | 0 | 1 | 1 |  |
| **BMI,** Mean (SD) | 28.3 (5.5) | 26.8 (4.9) | 28.1 (5.6) | 29.1 (5.4) | 29.0 (5.6) | <0.001 |
| Missing | 5 | 3 | 1 | 1 | 0 |  |
| **Diabetes, n (%)** | 43 (3.6%) | 4 (1.3%) | 3 (1.0%) | 12 (4.0%) | 24 (8.0%) | <0.001 |
| Missing | 1 | 0 | 1 | 0 | 0 |  |
| **Cardiovascular disease, n (%)** | 32 (2.7%) | 5 (1.7%) | 6 (2.0%) | 8 (2.7%) | 13 (4.3%) | 0.2 |
| **Non-melanoma cancers, n (%)** | 127 (11%) | 38 (13%) | 34 (11%) | 26 (8.7%) | 29 (9.7%) | 0.4 |
