# Supplementary material for: Association of epigenetic age acceleration with MRI biomarkers of aging and Alzheimer’s disease neurodegeneration
Source: Aging (Albany NY). 2026 Apr 7;18(1):303–26. doi: 10.18632/aging.206369 (PMC13285947; doi:10.18632/aging.206369)
Supplement: Supplementary Tables 2-8 [file aging-18-1-206369-s004.pdf]

Supplementary Table 2. Associations of each epigenetic clock with SPARE-BAA in progressively adjusted models.

Epigenetic clock	SD	Model	β (95% CI)	p
IEAA	5.05	1	-0.07 (-0.47, 0.34)	0.75
		2	-0.11 (-0.52, 0.29)	0.59
		3	-0.14 (-0.55, 0.27)	0.50
		4	-0.14 (-0.54, 0.27)	0.52
EEAA	6.15	1	-0.01 (-0.41, 0.40)	0.97
		2	-0.08 (-0.49, 0.33)	0.70
		3	-0.09 (-0.50, 0.31)	0.65
		4	-0.12 (-0.53, 0.30)	0.58
AgeAccelPheno	6.77	1	-0.01 (-0.41, 0.39)	0.95
		2	-0.05 (-0.45, 0.36)	0.82
		3	-0.08 (-0.48, 0.32)	0.70
		4	-0.19 (-0.63, 0.26)	0.41
DunedinPACE	0.10	1	0.17 (-0.24, 0.57)	0.42
		2	0.26 (-0.15, 0.66)	0.22
		3	0.19 (-0.23, 0.60)	0.38
		4	0.11 (-0.36, 0.58)	0.64
AgeAccelGrim2	4.15	1	0.34 (-0.06, 0.75)	0.10
		2	0.44 (0.04, 0.85)	0.03
		3	0.30 (-0.16, 0.76)	0.20
		4	0.38 (-0.17, 0.93)	0.18

Abbreviations: SD, standard deviation; IEAA, intrinsic epigenetic age acceleration; EEAA, extrinsic epigenetic age acceleration; AgeAccelPheno, phenotypic age acceleration; DunedinPACE, Dunedin Pace of Aging calculated from the Epigenome; AgeAccelGrim2, accelerated DNA methylation GrimAge version 2. Model 1 adjusted for age. Model 2 adjusted for age, race, and ethnicity. Model 3 adjusted for age, race, ethnicity, hormone trial arm, education, and smoking status. Model 4 adjusted for age, race, ethnicity, hormone trial arm, education, smoking status, physical activity, body mass index, diabetes, cardiovascular disease, non-melanoma cancer, and blood cell composition. For IEAA or EEAA, blood cell composition was not included in the fully adjusted model (see text).

Supplementary Table 3. Associations of each epigenetic clock with the ln(AD-PS+1) score.

Epigenetic clock	SD	Model	β (95% CI)	p
IEAA	5.05	1	-0.0017 (-0.0101, 0.0068)	0.70
		2	-0.0015 (-0.0100, 0.0070)	0.73
		3	-0.0024 (-0.0108, 0.0060)	0.58
		4	-0.0025 (-0.0109, 0.0059)	0.56
EEAA	6.15	1	0.0043 (-0.0041, 0.0128)	0.31
		2	0.0053 (-0.0032, 0.0139)	0.22
		3	0.0059 (-0.0026, 0.0144)	0.17
		4	0.0054 (-0.0031, 0.0140)	0.21
AgeAccelPheno	6.77	1	0.0045 (-0.0040, 0.0129)	0.30
		2	0.0048 (-0.0037, 0.0133)	0.27
		3	0.0038 (-0.0046, 0.0122)	0.37
		4	0.0040 (-0.0053, 0.0132)	0.40
DunedinPACE	0.10	1	0.0051 (-0.0034, 0.0135)	0.24
		2	0.0042 (-0.0044, 0.0127)	0.34
		3	0.0027 (-0.0059, 0.0113)	0.53
		4	0.0032 (-0.0066, 0.0131)	0.52
AgeAccelGrim2	4.15	1	0.0152 (0.0068, 0.0237)	<0.01
		2	0.0144 (0.0059, 0.0229)	<0.01
		3	0.0112 (0.0016, 0.0207)	0.02
		4	0.0151 (0.0035, 0.0266)	0.01

Abbreviations: SD, standard deviation; IEAA, intrinsic epigenetic age acceleration; EEAA, extrinsic epigenetic age acceleration; AgeAccelPheno, phenotypic age acceleration; DunedinPACE, Dunedin Pace of Aging calculated from the Epigenome; AgeAccelGrim2, accelerated DNA methylation GrimAge version 2. Model 1 adjusted for age. Model 2 adjusted for age, race, and ethnicity. Model 3 adjusted for age, race, ethnicity, hormone trial arm, education, and smoking status. Model 4 adjusted for age, race, ethnicity, hormone trial arm, education, smoking status, physical activity, body mass index, diabetes, cardiovascular disease, non-melanoma cancer, and blood cell composition. For IEAA or EEAA, blood cell composition was not included in the fully adjusted model (see text).

Supplementary Table 4. Associations of each epigenetic clock with the ln(AD-PS+1) score after excluding the 46 women with MCI/dementia at the MRI visit.

EpiAgeAccel	SD	Model	β (95% CI)	p
IEAA	5.04	1	-0.0013 (-0.0098, 0.0072)	0.76
		2	-0.0008 (-0.0093, 0.0078)	0.86
		3	-0.0015 (-0.0099, 0.0069)	0.73
		4	-0.0017 (-0.0101, 0.0068)	0.70
EEAA	6.11	1	0.0033 (-0.0052, 0.0117)	0.45
		2	0.0045 (-0.0040, 0.0130)	0.30
		3	0.0053 (-0.0031, 0.0138)	0.22
		4	0.0050 (-0.0035, 0.0135)	0.25
AgeAccelPheno	6.73	1	0.0049 (-0.0035, 0.0134)	0.25
		2	0.0055 (-0.0030, 0.0140)	0.20
		3	0.0048 (-0.0037, 0.0132)	0.27
		4	0.0054 (-0.0038, 0.0146)	0.25
DunedinPACE	0.10	1	0.0040 (-0.0044, 0.0125)	0.35
		2	0.0033 (-0.0052, 0.0118)	0.45
		3	0.0019 (-0.0066, 0.0105)	0.66
		4	0.0016 (-0.0082, 0.0114)	0.75
AgeAccelGrim2	4.14	1	0.0158 (0.0074, 0.0243)	<0.01
		2	0.0150 (0.0065, 0.0236)	<0.01
		3	0.0124 (0.0029, 0.0220)	0.01
		4	0.0163 (0.0048, 0.0277)	<0.01

Abbreviations: SD, standard deviation; IEAA, intrinsic epigenetic age acceleration; EEAA, extrinsic epigenetic age acceleration; AgeAccelPheno, phenotypic age acceleration; DunedinPACE, Dunedin Pace of Aging calculated from the Epigenome; AgeAccelGrim2, accelerated DNA methylation GrimAge version 2. Model 1 adjusted for age. Model 2 adjusted for age, race, and ethnicity. Model 3 adjusted for age, race, ethnicity, hormone trial arm, education, and smoking status. Model 4 adjusted for age, race, ethnicity, hormone trial arm, education, smoking status, physical activity, body mass index, diabetes, cardiovascular disease, non-melanoma cancer, and blood cell composition. For IEAA or EEAA, blood cell composition was not included in the fully adjusted model (see text).

Supplementary Table 5. Associations of each epigenetic clock with SPARE-BAA stratified by APOE ε4 carrier status.

EpiAgeAccel	Model	Carrier		Non-carrier		P interaction
		SD	β (95% CI)	SD	β (95% CI)	
IEAA	1	4.71	0.60 (-0.34, 1.53)	5.14	-0.29 (-0.77, 0.19)	0.09
	2		0.54 (-0.40, 1.47)		-0.33 (-0.81, 0.15)	0.10
	3		0.56 (-0.39, 1.51)		-0.31 (-0.79, 0.17)	0.11
EEAA	1	6.39	-0.28 (-1.23, 0.67)	6.02	0.07 (-0.41, 0.55)	0.59
	2		-0.35 (-1.31, 0.60)		0.06 (-0.42, 0.54)	0.52
	3		-0.33 (-1.31, 0.65)		0.02 (-0.47, 0.50)	0.57
AgeAccelPheno	1	6.62	-0.23 (-1.17, 0.71)	6.77	0.01 (-0.47, 0.49)	0.72
	2		-0.42 (-1.38, 0.54)		-0.02 (-0.50, 0.46)	0.66
	3		0.00 (-1.05, 1.05)		-0.26 (-0.79, 0.26)	0.78
DunedinPACE	1	0.11	0.07 (-0.87, 1.01)	0.10	0.38 (-0.10, 0.86)	0.56
	2		-0.08 (-1.06, 0.90)		0.33 (-0.16, 0.82)	0.56
	3		-0.23 (-1.33, 0.87)		0.22 (-0.34, 0.78)	0.45
AgeAccelGrim2	1	3.96	0.13 (-0.81, 1.06)	4.05	0.58 (0.10, 1.06)	0.37
	2		-0.37 (-1.48, 0.73)		0.52 (-0.02, 1.07)	0.30
	3		0.08 (-1.26, 1.42)		0.48 (-0.18, 1.13)	0.30

Abbreviations: SD, standard deviation; IEAA, intrinsic epigenetic age acceleration; EEAA, extrinsic epigenetic age acceleration; AgeAccelPheno, phenotypic age acceleration; DunedinPACE, Dunedin Pace of Aging calculated from the Epigenome; AgeAccelGrim2, accelerated DNA methylation GrimAge version 2. Model 1 adjusted for age. Model 2 adjusted for age, race, and ethnicity. Model 3 adjusted for age, race, ethnicity, hormone trial arm, education, and smoking status. Model 4 adjusted for age, race, ethnicity, hormone trial arm, education, smoking status, physical activity, body mass index, diabetes, cardiovascular disease, non-melanoma cancer, and blood cell composition. For IEAA or EEAA, blood cell composition was not included in the fully adjusted model (see text).

P-interactions were obtained from likelihood ratio test comparing the main effects model with a model including the interaction term between scaled epigenetic clock and APOE ε4 carrier status.

Supplementary Table 6. Associations of each epigenetic clock with the ln(AD-PS+1) score stratified by APOE ε4 carrier status.

EAA	Model	Carrier		Non-carrier		P interaction
		SD	β (95% CI)	SD	β (95% CI)	
IEAA	1	4.71	0.0017 (-0.0188, 0.0222)	5.14	-0.0001 (-0.0102, 0.0099)	0.87
	2		0.0003 (-0.0204, 0.0209)		-0.0013 (-0.0113, 0.0086)	0.81
	3		0.0001 (-0.0209, 0.0211)		-0.0008 (-0.0108, 0.0091)	0.88
EEAA	1	6.39	0.0162 (-0.0044, 0.0369)	6.02	0.0034 (-0.0066, 0.0135)	0.30
	2		0.0167 (-0.0042, 0.0377)		0.0039 (-0.0061, 0.0138)	0.28
	3		0.0169 (-0.0045, 0.0383)		0.0028 (-0.0072, 0.0128)	0.25
AgeAccelPheno	1	6.62	0.0011 (-0.0195, 0.0217)	6.77	0.0073 (-0.0028, 0.0173)	0.57
	2		-0.0014 (-0.0225, 0.0197)		0.0058 (-0.0042, 0.0157)	0.69
	3		-0.0015 (-0.0255, 0.0224)		0.0047 (-0.0061, 0.0155)	0.79
DunedinPACE	1	0.11	0.0015 (-0.0191, 0.0221)	0.10	0.0040 (-0.0061, 0.0141)	0.80
	2		-0.0042 (-0.0257, 0.0172)		0.0032 (-0.0069, 0.0133)	0.75
	3		-0.0074 (-0.0325, 0.0177)		0.0032 (-0.0084, 0.0148)	0.65
AgeAccelGrim2	1	3.96	0.0196 (-0.0008, 0.0400)	4.05	0.0131 (0.0030, 0.0232)	0.53
	2		0.0111 (-0.0132, 0.0353)		0.0099 (-0.0013, 0.0212)	0.57
	3		0.0112 (-0.0193, 0.0418)		0.0129 (-0.0006, 0.0264)	0.54

Abbreviations: SD, standard deviation; IEAA, intrinsic epigenetic age acceleration; EEAA, extrinsic epigenetic age acceleration; AgeAccelPheno, phenotypic age acceleration; DunedinPACE, Dunedin Pace of Aging calculated from the Epigenome; AgeAccelGrim2, accelerated DNA methylation GrimAge version 2. Model 1 adjusted for age. Model 2 adjusted for age, race, and ethnicity. Model 3 adjusted for age, race, ethnicity, hormone trial arm, education, and smoking status. Model 4 adjusted for age, race, ethnicity, hormone trial arm, education, smoking status, physical activity, body mass index, diabetes, cardiovascular disease, non-melanoma cancer, and blood cell composition. For IEAA or EEAA, blood cell composition was not included in the fully adjusted model (see text).

P-interactions were obtained from likelihood ratio test comparing the main effects model with a model including the interaction term between scaled epigenetic clock and APOE ε4 carrier status.

Supplementary Table 7. Associations of each epigenetic clock with SPARE-BAA adjusted for time interval since baseline.

Epigenetic clock	SD	Model	β (95% CI)	p
IEAA	5.05	1	-0.06 (-0.46, 0.34)	0.78
		2	-0.10 (-0.51, 0.30)	0.62
		3	-0.13 (-0.54, 0.28)	0.53
		4	-0.13 (-0.53, 0.28)	0.55
EEAA	6.15	1	-0.03 (-0.44, 0.37)	0.88
		2	-0.10 (-0.51, 0.31)	0.62
		3	-0.11 (-0.52, 0.30)	0.59
		4	-0.14 (-0.55, 0.28)	0.52
AgeAccelPheno	6.77	1	-0.04 (-0.44, 0.37)	0.86
		2	-0.07 (-0.47, 0.34)	0.74
		3	-0.10 (-0.51, 0.31)	0.63
		4	-0.19 (-0.63, 0.26)	0.41
DunedinPACE	0.10	1	0.14 (-0.26, 0.55)	0.48
		2	0.24 (-0.17, 0.65)	0.26
		3	0.17 (-0.25, 0.58)	0.43
		4	0.11 (-0.36, 0.58)	0.65
AgeAccelGrim2	4.15	1	0.32 (-0.09, 0.72)	0.13
		2	0.42 (0.01, 0.83)	0.05
		3	0.27 (-0.19, 0.74)	0.25
		4	0.37 (-0.18, 0.92)	0.19

Abbreviations: SD, standard deviation; IEAA, intrinsic epigenetic age acceleration; EEAA, extrinsic epigenetic age acceleration; AgeAccelPheno, phenotypic age acceleration; DunedinPACE, Dunedin Pace of Aging calculated from the Epigenome; AgeAccelGrim2, accelerated DNA methylation GrimAge version 2. Model 1 adjusted for age and time between blood draw and MRI assessment. Model 2 adjusted for age, time interval between blood draw and MRI assessment, race, and ethnicity. Model 3 adjusted for age, time interval between blood draw and MRI assessment, race, ethnicity, hormone trial arm, education, and smoking status. Model 4 adjusted for age, time interval between blood draw and MRI assessment, race, ethnicity, hormone trial arm, education, smoking status, physical activity, body mass index, diabetes, cardiovascular disease, non-melanoma cancer, and blood cell composition. For IEAA or EEAA, blood cell composition was not included in the fully adjusted model (see text).

Supplementary Table 8. Associations of each epigenetic clock with the ln(AD-PS+1) score adjusted for time interval since baseline.

Epigenetic clock	SD	Model	β (95% CI)	p
IEAA	5.05	1	-0.0020 (-0.0104, 0.0064)	0.64
		2	-0.0020 (-0.0105, 0.0065)	0.65
		3	-0.0030 (-0.0114, 0.0055)	0.49
		4	-0.0031 (-0.0115, 0.0054)	0.48
EEAA	6.15	1	0.0054 (-0.0030, 0.0139)	0.21
		2	0.0064 (-0.0021, 0.0150)	0.14
		3	0.0071 (-0.0014, 0.0155)	0.10
		4	0.0066 (-0.0019, 0.0151)	0.13
AgeAccelPheno	6.77	1	0.0056 (-0.0028, 0.0141)	0.19
		2	0.0059 (-0.0026, 0.0144)	0.17
		3	0.0050 (-0.0034, 0.0134)	0.24
		4	0.0040 (-0.0052, 0.0132)	0.39
DunedinPACE	0.10	1	0.0061 (-0.0023, 0.0146)	0.16
		2	0.0053 (-0.0033, 0.0138)	0.23
		3	0.0039 (-0.0047, 0.0125)	0.38
		4	0.0034 (-0.0064, 0.0132)	0.50
AgeAccelGrim2	4.15	1	0.0169 (0.0085, 0.0254)	<0.01
		2	0.0161 (0.0075, 0.0247)	<0.01
		3	0.0132 (0.0036, 0.0228)	<0.01
		4	0.0155 (0.0041, 0.0270)	<0.01

Abbreviations: SD, standard deviation; IEAA, intrinsic epigenetic age acceleration; EEAA, extrinsic epigenetic age acceleration; AgeAccelPheno, phenotypic age acceleration; DunedinPACE, Dunedin Pace of Aging calculated from the Epigenome; AgeAccelGrim2, accelerated DNA methylation GrimAge version 2. Model 1 adjusted for age and time between blood draw and MRI assessment. Model 2 adjusted for age, time interval between blood draw and MRI assessment, race, and ethnicity. Model 3 adjusted for age, time interval between blood draw and MRI assessment, race, ethnicity, hormone trial arm, education, and smoking status. Model 4 adjusted for age, time interval between blood draw and MRI assessment, race, ethnicity, hormone trial arm, education, smoking status, physical activity, body mass index, diabetes, cardiovascular disease, non-melanoma cancer, and blood cell composition. For IEAA or EEAA, blood cell composition was not included in the fully adjusted model (see text).
